# Classic Psychedelics in Pain Modulation: Mechanisms, Clinical Evidence, and Future Perspectives

**DOI:** 10.1021/acschemneuro.5c00152

**Published:** 2025-06-06

**Authors:** Anna Czopek, Jakub Jończyk, Monika Fryc, Daria Kluzik, Agnieszka Zagórska

**Affiliations:** Department of Medicinal Chemistry, Faculty of Pharmacy, 49573 Jagiellonian University Medical College, Medyczna 9, Kraków 30-688, Poland

**Keywords:** classic psychedelics, chronic pain, psilocybin, LSD, serotonin 5-HT_2A_ receptors

## Abstract

Millions worldwide suffer from chronic pain, a complex condition often accompanied by depression and anxiety, highlighting the urgent need for innovative treatments. Classic psychedelics, including psilocybin, lysergic acid diethylamide (LSD), and *N*,*N*-dimethyltryptamine (DMT), primarily act on serotonin 5-HT_2A_ receptors and have emerged as potential modulators of pain perception and mood regulation. These substances may offer an alternative to conventional analgesics, such as opioids and nonsteroidal anti-inflammatory drugs (NSAIDs), by influencing neuroplasticity, descending pain modulation pathways, and inflammatory processes. Evidence from case studies, preclinical research, and early phase clinical trials suggests that psychedelics may alleviate pain in conditions such as cluster headaches, migraines, fibromyalgia, and chronic pain syndromes. However, the exact mechanisms underlying their analgesic properties are yet to be fully understood. While psychedelics show promise in reshaping pain management strategies, rigorous randomized controlled trials are needed to establish their safety, efficacy, and optimal dosing. This review highlights the therapeutic potential of psychedelics for chronic pain and emphasizes the necessity of further research to validate their role in modern pain medicine.

## Graphical abstract

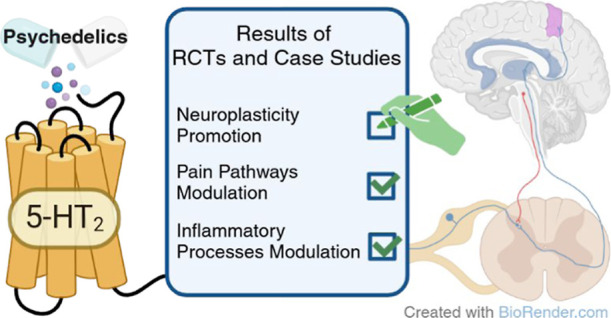

## 1 Introduction

In 2020, the International Association for the Study of Pain (IASP) introduced a revised definition of pain for the first time since 1979. The IASP defines pain as an unpleasant sensory and emotional experience associated with or resembling that associated with, actual or potential tissue damage.^1^ This definition emphasizes that pain is both a physical sensation and an emotional response, making it a highly subjective experience. The definition serves as a key protective mechanism, alerting the body to potential harm and prompting actions to prevent or minimize further injury.

Pain is a complex physiological and psychological phenomenon that can be classified based on several criteria, including pathophysiology, duration, origin, and etiology. Understanding these classifications is essential for accurate diagnosis and effective management.

Pathophysiology-based classification categories pain according to its underlying mechanism.^2^ Nociceptive pain arises from activating nociceptors in response to actual or potential tissue damage. It can be further divided into somatic pain, affecting skin, muscles, bones, and connective tissues, and visceral pain, originating from internal organs. Neuropathic pain results from nerve damage or dysfunction, commonly seen in diabetic neuropathy and postherpetic neuralgia, whereas inflammatory pain results from immune system activation, as in rheumatoid arthritis. Pain may be acute, with a sudden onset and short duration, while chronic pain persists beyond three to six months and often lacks an identifiable cause. Specific causes of pain include cancer pain, postoperative pain, musculoskeletal pain, and psychogenic pain, which have no identifiable physical origin. Pain can also occur in distinct conditions, such as central pain syndrome, phantom limb pain, and headache disorders. Proper classification is crucial for guiding effective treatment strategies.^3^

Classic psychedelics, such as psilocybin, lysergic acid diethylamide (LSD), and *N*,*N*-dimethyltryptamine (DMT), primarily activate the serotonin 5-HT_2_ receptors, particularly 5-HT_2A_ receptors and have emerged as potential therapeutic agents for managing chronic pain.^4^ While traditional pain management strategies often rely on opioids or nonsteroidal anti-inflammatory drugs (NSAIDs), psychedelics offer a novel approach that targets both the physical and psychological dimensions of pain. Given the high comorbidity of chronic pain with depression and anxiety, psychedelics’ ability to enhance mood and alter pain perception makes them a promising alternative in pain management. Ongoing research and clinical trials aim to clarify their safety and efficacy in this context.^5^

Classic psychedelics reviews in pain management were the subject of 13 review papers published between 2020 and 2024 (PubMed).^6–18^ This data set compiles key publications on the therapeutic applications of psychedelic substances such as LSD, psilocybin, and cannabinoids in the treatment of chronic and nociplastic pain. It features studies examining neuroimaging, expectancy effects, and clinical outcomes. The reviews explore psychoactive and nonintoxicating compounds, focusing on their analgesic mechanisms and psychopharmacological profiles. However, these findings remain dispersed across disciplines and types of studies, with no comprehensive synthesis focusing specifically on their mechanism of action and therapeutic potential in pain treatment. While reviews highlight clinical and neuropsychological aspects of psychedelics in pain management, they largely omit detailed discussions on the pharmacokinetics, metabolism, and bioavailability of these substances, which are crucial for understanding their therapeutic potential and optimizing their efficacy. Moreover, mechanistic insights into receptor binding affinities downstream signaling pathways (e.g., 5-HT_2A_ vs nonclassical targets) are absent or minimally addressed.

This review aims to address this gap by collating and analyzing the most recent evidence on use of classic psychedelics in pain modulation, while integrating insights into their molecular pharmacology, clinical applications, and underlying neurobiological mechanisms, particularly those involving the 5-HT_2A_ receptor. The novelty of the review is reflected in several key areas. This manuscript offers an integrative analysis of both G-protein and β-arrestin pathways activated via 5-HT_2A_ receptor signaling and their relevance to synaptic plasticity and pain neurocircuitry remodeling. It further examines interactions with BDNF–TrkB and dopaminergic systems, offering novel insights into nonopioid analgesia. In addition, the pharmacokinetics of classical psychedelics are characterized to contextualize their therapeutic time course and inform dosing considerations. The review synthesizes findings from recent clinical trials, case studies, and preclinical research focusing on classic psychedelic efficacy in pain management associated with conditions such as cluster headaches, migraines, fibromyalgia, and chronic pain syndromes. Methodological challenges in clinical trials involving psychedelic substances, such as difficulties with blinding and the influence of expectation bias, are critically assessed, with recommendations offered to enhance the rigor of future trials. Overall, this review underscores the importance of continued research into psychedelic-assisted therapies as a novel and promising approach to the treatment of both acute and chronic pain.

## 2 Basic Mechanisms of Pain Processing: Transduction, Transmission, Modulation, and Perception

Pain perception is a series of sensory events that allow the brain to recognize and respond to potential threats.^19^ Understanding these processes is essential for revealing how serotonergic modulation—particularly via 5-HT_2A_ receptors—affects pain signaling and perception. Psychedelics such as LSD or psilocybin are thought to influence the central nervous system primarily through these receptors, modulating how pain is processed and experienced.

The pain pathway theory explains how nociceptive signals are generated, transmitted, and processed within the nervous system, ultimately producing the sensation of pain.^3^ Figure 1 illustrates this process regulating pain perception, response, and experience.

**Figure 1:**
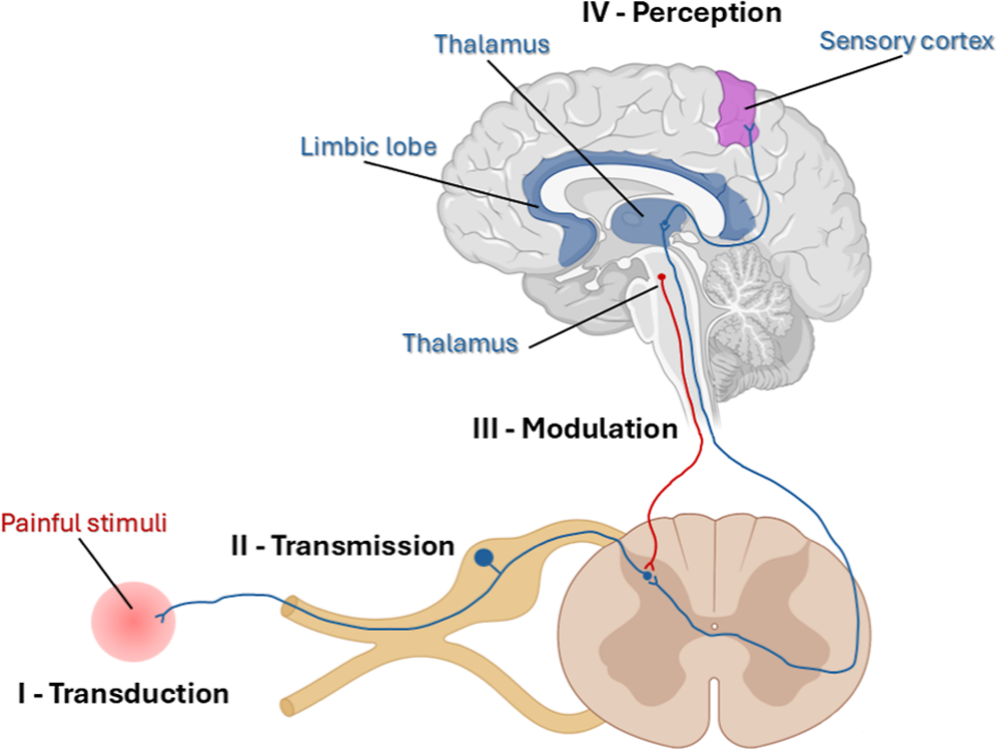
Illustration of the pain transmission pathway with four stages of nociception—transduction, transmission, modulation, and perception—within the ascending (blue) and descending (red) neural pathways. Peripheral nociceptors initiate transduction (I) by converting noxious mechanical, thermal, or chemical stimuli into electrical signals.^20^ The transmission (II) of these impulses occurs via primary afferent neurons to the spinal cord’s dorsal horn, subsequently reaching higher brain centers.^21^ The modulation (III) of nociceptive signals is achieved primarily through descending pathways originating in the brainstem (e.g., the periaqueductal gray (PAG) and rostroventral medulla (RVM)), where neurotransmitters—serotonin, norepinephrine, and endogenous opioids—mediate either the enhancement or the suppression of nociceptive transmission.^22,23^ Conscious pain perception (IV) arises from the cortical integration of nociceptive input with its emotional and cognitive context.^24,25^ At multiple levels, particularly in modulation (III) and perception (IV), serotonergic activity—mediated in part through 5-HT_2_A receptor signaling—critically influences pain intensity and emotional perception. Created with BioRender.

Specifically, 5-HT_2A_R have been identified as key modulators of both central pain processing and chronic inflammatory states.^26^ These receptors, densely expressed on cortical glutamatergic excitatory pyramidal neurons and to a lesser extent on GABAergic interneurons, exert complex effects depending on their anatomical location.^27^ The role of 5-HT_2A_R in pain modulation varies depending on the type of pain (acute or chronic) and the location of the receptors. In the PNS, 5-HT_2A_R activation is pro-inflammatory, contributing to inflammatory pain, whereas in the CNS, it has an antinociceptive effect, helping to reduce pain perception.^28^

## 3 Classic Psychedelics: Structure and Mechanisms of Action

### 3.1 –Mechanism of Action of Classic Psychedelics

The effects of psychedelics are a result of complex and interconnected influences on the brain at the molecular, cellular level, circuit and brain network, as well. At the molecular and cellular level, psychedelics primarily activate the 5-HT_2_Rs along with other serotonin subreceptors, tropomyosin receptor kinase B (TrkB), and dopamine receptors.^29^

The 5-HT_2_Rs, a class of G-protein-coupled receptor (GPCR), are naturally activated by its endogenous ligand, serotonin (5-HT), which acts as an agonist. Activation of this receptor by psychedelic drugs causes significant changes in perception and cognition. The cubic ternary complex model effectively illustrates how the dynamic and complex nature of GPCR signal transduction leads to these effects.^30,31^ According to this model, ligand binding shifts the equilibrium between distinct receptor conformations, resulting in a signaling “bias” favoring either G-protein-dependent or β-arrestin-dependent pathways. The stimulation of Gq-like G proteins by psychedelics leads to the activation of phospholipase C gamma (PLCγ), resulting in intracellular calcium release via inositol trisphosphate (IP_3_) and diacylglycerol (DAG)-mediated protein kinase C (PKC) activation. These signaling events subsequently trigger multiple downstream pathways, including the extracellular signal-regulated kinases (ERKs), cyclic adenosine monophosphate (cAMP) response element-binding protein (CREB), and mammalian target of rapamycin (mTOR) pathways, which influence neuronal activity. The influx of calcium and the activation of calmodulin-dependent protein kinase II and IV (CaMKII and CaMKIV) further enhance synaptic plasticity by interacting with *N*-methyl- *d* -aspartate receptor (NMDA)-type glutamate receptors and phosphorylating α-amino-3-hydroxy-5-methyl-4-isoxazolepropionic acid receptor (AMPA)-type glutamate receptor subunits. This synaptic strengthening supports long-term potentiation (LTP), a key process in learning and memory. Beyond G-protein-mediated signaling, 5-HT_2A_R agonism can activate β-arrestin-dependent pathways via phosphoinositide 3-kinases and protein kinase B. Both G-protein- and β-arrestin-mediated signaling contribute to neural plasticity and may underline the sustained therapeutic effects of psychedelics in psychiatric disorders (Figure 2).^32,33^

**Figure 2:**
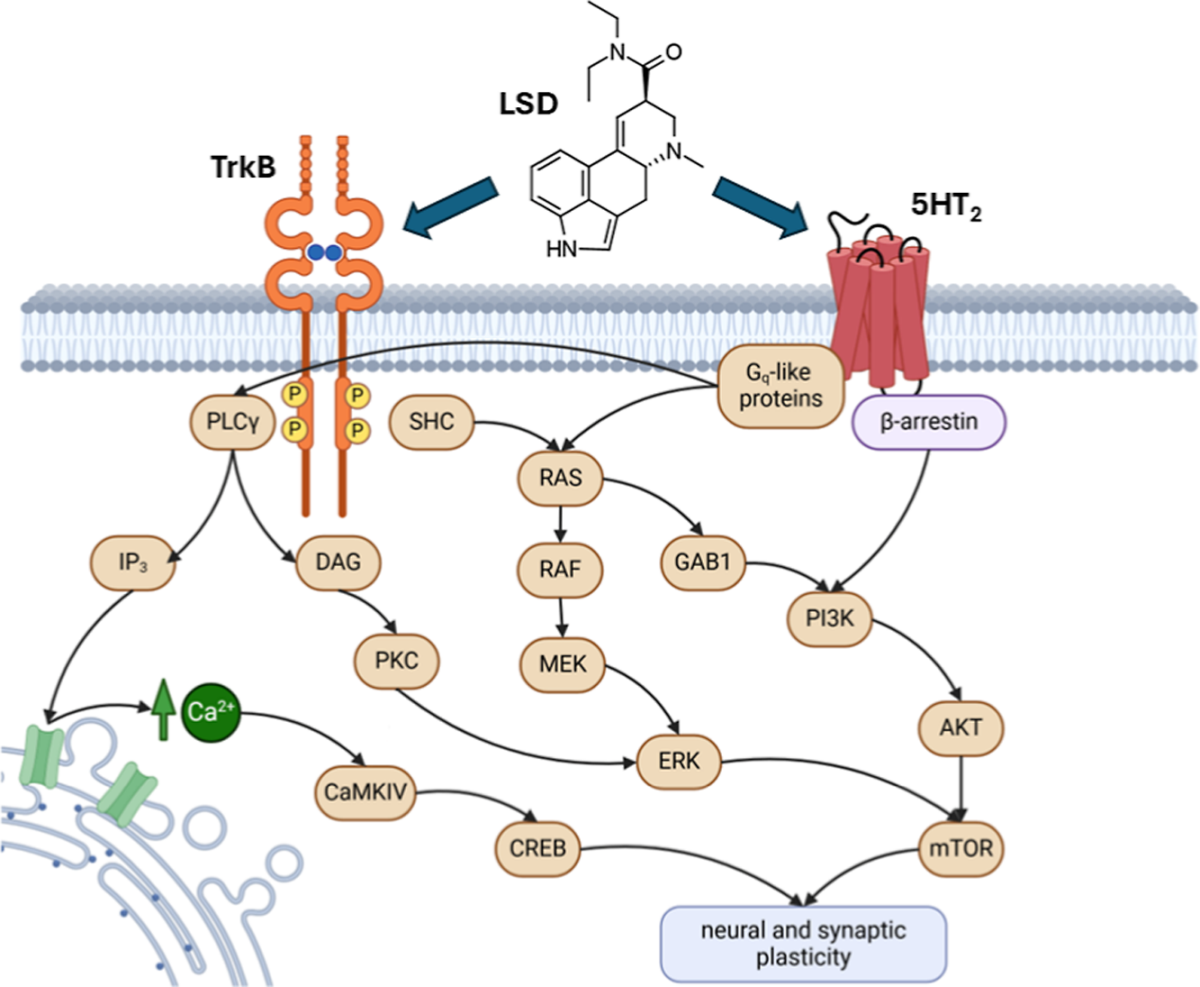
Diagram illustrates the downstream signaling cascades initiated by LSD binding to 5-HT_2_Rs and TrkB receptors. Created in BioRender.

Until the end of the 20th century, classic psychedelics were believed to act primarily through 5-HT_2A_ serotonin receptors. However, it is now known that psychedelics interact with multiple serotonin receptors (5-HTRs), including 5-HT_1A_, 5-HT_2A_, 5-HT_2B_, and 5-HT_2C_ LSD and psilocin have the highest affinity for 5-HT_2A–C_ receptors (Table 1). In contrast, mescaline has the lowest, which corresponds to the significantly higher therapeutic dose required to achieve its effects (Table 2). It is noteworthy that all substances listed in the table function as agonists or partial agonists of the 5-HT_2A_R, which is coupled with both the Gq signaling pathway and β-arrestin recruitment. Recent studies have indicated that 5-HT_2A_ Gq receptor agonists exhibiting an Emax greater than 70% are typically associated with psychedelic effects, distinguishing them from nonpsychedelic 5-HT_2A_R agonists. These findings suggest that.

**Table 1:** Affinity and Intrinsic Activity of Classical Psychedelic Substances, Including LSD, Psilocybin, DMT, and Mescaline, for Serotonergic (5-HT_1A_, 5-HT_2A–C_), Dopaminergic (D_1_, D_2_), Adrenergic (α_1A_), Histaminergic (H_1_) and Tropomyosin Kinase B (TrkB) Receptors^a^

receptors	compound			
*K*_i_ [nM]/EC_50_ or *K*_b_ [*n*M]	LSD	psilocin	DMT	mescaline
5-HT_1A_	3–7.3/*A [1.31] **PA [187]	62.6–567/PA [1.7]	>75/A [210]	1841–4600/-
5-HT_2A_	4–11.3/*A [0.35] **A [0.82]	49–339.6/*PA [19.7] **PA [43.5]	237–2323/*PA [172] **PA [117]	>6000/*PA [645] **PA [426]
5-HT_2B_	30/*A [0.3] **PA [1.51]	4.7/PA [21.5]	107.6/-	792.8/*PA [1096]
5-HT_2C_	15–30.6/*A [0.66] **PA [3.92]	10–141.2/PA [136]	334.6–2000/A [104]	>6000/*PA [316]
TrkB	3.38/-	6.73/-	-/-	-/-
D_1_	177/AN [841.03]	19.9/AN	271.1/AN	-/-
D_2_	110.1/A [2.17]	-/-	-/-	-/-
α_1A_	1127.1/AN	-/-	1745/AN	-/-
H_1_	1543.5/AN	-/-	-/-	-/-

a - not measured, * value for G pathway, ** value for β-arrestin pathway, A–agonist; PA–partial agonist; AN–antagonist. Receptors affinities were taken from T.S.Ray, Ricki et al., Cumming et al., Erkizia-Santamaria et al., Moliner et al., Wallach et al., Thomann et al., Kozell et al., Fan et al^29,34,38–44^ .

**Table 2:**
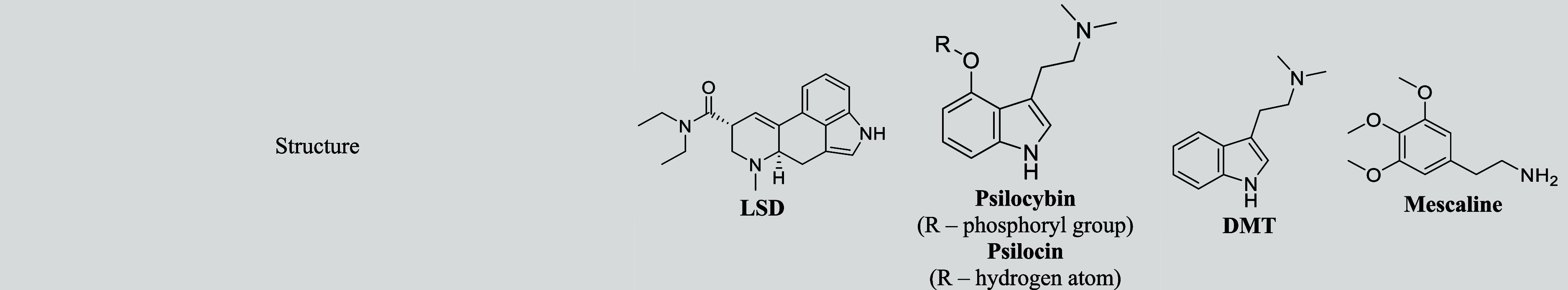
Pharmacokinetic Properties of Classic Psychedelics, Including LSD, Psilocybin, DMT, and Mescaline^a^

compound chemical name	logP, logS	BBB permeant	pharmacokinetic properties				
			*C*_max_ [ng/mL]	*T*_max_ [h]	*T*_1/2_ [h]	CL/F [L\h]	d.e [h]
LSD (6aR,9R)-*N*,*N*-diethyl-7-methyl-6,6a,8,9-tetrahydro-4H-indolo[4,3-fg]quinoline-9-carboxamide	2.76, −3.72	yes	1.1–3.6	0.5–3.5	2.3–4.8	19–62	8.2
psilocybin/Psilocin* [3-[2-(dimethylamino)ethyl]-1H-indol-4-yl] dihydrogen phosphate/3-(2-(dimethylamino)ethyl)-1H-indol-4-ol	0.47, −0.62/1.95, −2.68	no/yes	9.6–34	1–5	1.5–2.9	259–938	4.9
DMT 2-(1H-indol-3-yl)-*N*,*N*-dimethylethanamine	2.15, −2.34	yes	11–107	0.03–0.16	0.04–0.18	1320–3660	0.3
mescaline 2-(3,4,5-trimethoxy-phenyl)ethanamine	1.43, −1.61	yes	721–1822	1.5–4	2.6–4.3	143–412	11.1

a LogP logS and BBB permeant parameters were predicted using www.swissadme.ch, pharmacokinetics parameters of LSD (100 μg dose o.a.), psilocybin (20 mg dose o.a.), mescaline (500 mg dose o.a.) were taken from L.Lay et al.,^56^ pharmacokinetics parameters of DMT (25 mg bolus+1 mg/min by 90 min) were taken from S.B.Vogt et al.,^55^ *C*_max_–maximum concentration, *T*_max_–time to maximum, *T*_1/2_–terminal elimination half-time, CL/F- clearance, d.e-duration effect; *pharmacokinetics properties were reported for psi.

Gq-mediated efficacy at the 5-HT_2A_ receptor is essential for the induction of psychedelic effects.^34^ Additionally, the agonistic activity at the 5-HT_2B_R exhibited by all psychedelics listed in the table has been associated with a risk of cardiac valvulopathy, particularly in chronic use.^35^ Beyond their strong affinity for 5-HTRs, classic psychedelics also influence other receptor systems. For example, LSD interacts with dopamine (D_1_R and D_2_R), adrenergic (α_1_R) and histaminergic (H_1_R) receptors, which can lead to cardiovascular side effects. Similarly, psilocin strongly activates 5-HTRs and interacts with dopamine (D_1_R) receptors. Although psilocin and LSD primarily interact through 5-HT_2A_R activation, their interaction with D_1_R and D_2_R also plays a crucial role. LSD binds directly to D_1_R and D_2_R, enhancing mood through dopamine release in the mesolimbic pathway. In contrast, while not strongly binding to D_2_R, psilocin indirectly increases dopaminergic activity, influencing reward processing and emotional responses. These interactions suggest that dopamine signaling contributes significantly to the euphoric effects of classic psychedelics.^36–38^

For their potential to modulate synaptic plasticity, which may bridge the therapeutic gap in analgesia. Synaptic plasticity is the ability of synapses to strengthen or weaken over time in response to activity. This phenomenon is fundamental to neural circuit modification and functional connectivity. Dendrites, the branching extensions of neurons, integrate electrochemical signals through numerous small protrusions known as spines, which receive inputs from individual axons.

Recent studies have demonstrated that psychedelics can promote structural and functional neural plasticity. For instance, compounds such as LSD and DMT have been shown to increase dendritic arbor complexity, promote dendritic spine growth, and stimulate synapse formation.^45^ The promotion of synaptic plasticity by psychedelics involves the activation of 5-HT_2A_Rs, leading to the release of the brain-derived neurotrophic factor (BDNF) and TrkB signaling pathway. This cascade facilitates the growth and strengthening of synaptic connections, thereby enhancing neural network adaptability.^46^ Recent findings demonstrate that classic psychedelics such as LSD directly bind to the TrkB receptor and enhance BDNF signaling.^29^ Thus, neuroplasticity enhancement is one of the primary mechanisms by which psychedelics may alleviate pain. These compounds promote synaptic remodeling and dendritic growth, potentially reversing maladaptive pain circuits in chronic pain conditions such as fibromyalgia, complex regional pain syndrome, and phantom limb pain.^47^ Furthermore, psychedelics influence the default mode network, which plays a key role in self-referential thinking and pain-related rumination. By disrupting maladaptive network connectivity, these substances may reduce the emotional distress associated with chronic pain.^48,49^ Additionally, psychedelics exhibit anti-inflammatory properties, reducing the release of pro-inflammatory cytokines such as interleukine-6 (IL-6) and tumor necrosis factor α (TNF-α), which are implicated in neuropathic and inflammatory pain conditions.^50^ Preclinical studies suggest that psychedelics also modulate glial cell activity, further contributing to pain relief.^51^

### 3.2 Characterization of Classic Psychedelics

Classic psychedelics, such as LSD, psilocybin, DMT, and mescaline, are derivatives of different (hetero)­cyclic systems and exhibit distinct physicochemical properties that influence their bioavailability, blood–brain barrier (BBB) permeability, and pharmacokinetics. LSD, an ergoline derivative, is the most lipophilic (Table 2) and efficiently crosses the BBB, resulting in a prolonged duration.^41^ Psilocybin, a tryptamine derivative and a highly water-soluble prodrug (Table 2) requires enzymatic conversion to psilocin, the active compound responsible for its psychoactive effects. Psilocin is slightly less lipophilic than LSD but still crosses the BBB effectively. Its impact on the CNS is shorter than those of LSD due to its shorter half-life.^52,53^ A clinical trial (NCT04673383) showed that the tryptamine derivative DMT, though capable of rapid BBB permeability, has poor oral bioavailability due to monoamine oxidase (MAO) metabolism, requiring MAO inhibitors for prolonged effects.^54^ Following intravenous administration, DMT reaches its peak effects within 2 to 10 min, with the total duration of its psychoactive effects lasting approximately 20 min before rapidly diminishing.^55^ In clinical trial (NCT04227756), LSD, psilocin, and mescaline were compared, with the latter exhibiting moderate lipophilicity and requiring higher doses (300–500 mg) to achieve psychoactive effects. Mescaline, a phenethylamine derivative, has the most extended duration of effects (Table 2), followed by LSD and psilocybin, due to its more prolonged time to reach peak plasma concentrations.^56^ Additionally, among the psychedelics listed in Table 2, LSD has the longest half-life and the lowest clearance, suggesting a slow elimination process and prolonged presence of the drug in the bloodstream. This may contribute to its extended duration of action compared to mainly psilocin and DMT. Differences in lipophilicity, solubility, BBB permeability and metabolism explain the varying onset and duration effect seen in classic psychedelics listed in Table 2.

## 4 Classic Psychedelics in Pain Management

Despite the lack of a complete understanding of the pain-modulating mechanism of psychedelics, psilocybin and LSD have shown promising effects in treating acute pain disorders such as cluster headaches and migraine, as well as chronic conditions such as fibromyalgia and low back pain.

The following literature review is based on a comprehensive search conducted using databases such as PubMed and the ClinicalTrials.gov Web site, focusing on case reports, surveys, clinical trials, observational studies, and reviews. Studies from the past 10 years were included to capture recent developments and ongoing challenges. Key search terms included as “psilocybin,” or “lysergic acid diethylamide,” or “*N*,*N*-dimethyltryptamine,” or “mescaline,” and “pain”. Based on these criteria, 38 clinical trials and 59 articles were initially identified. All trial summaries and article abstracts were reviewed, and duplicates, withdrawn trials, and studies focusing on conditions other than pain (e.g., depression, addiction, etc.) were excluded. The review below presents 20 full-text articles and the published results of 6 clinical trials.

### 4.1 Cluster Headache and Migraine

According to the International Classification of Headache Disorders, cluster headaches (CHs) and migraines are classified as primary headache disorders.^57^ While both are severe headache disorders, they differ in their characteristics, symptoms, and impact on daily life.

CHs are rare, affecting about 0.1% of the population, and are more common in men. They cause excruciating, burning, or piercing pain around one eye or on one side of the head, lasting 15 min to 3 h. CHs occur in cycles, with multiple attacks over weeks or months, often triggered by alcohol, strong odors, or sleep changes. CH is the most prevalent form of trigeminal autonomic cephalalgias (TACs), a group of headache disorders and is classified into two subtypes: episodic and chronic. The exact cause of CH is currently unknown. CHs are considered one of the most painful conditions, occurring multiple times a day during a cluster period. Due to their intensity and frequency, cluster headaches require rapid-relief treatments. Acute treatments effectively relieve pain during attacks, while prophylactic therapies are designed to reduce the frequency of daily episodes. Currently, there is no curative treatment for cluster headaches. The European Federation of Neurological Societies (EFNS) has established guidelines, including evidence-based recommendations and best practice points, for the management of CH.^58^ Current treatments include oxygen therapy, triptans, and preventative medications like verapamil, but these options often provide incomplete relief or come with side effects.^59^

Migraines, affecting 12% of the population (more common in women), cause throbbing pain on one or both sides of the head, lasting from 4 to 72 h. Migraines vary in intensity from moderate to severe and can last for days. They often come with nausea, vomiting, and sensitivity to light and sound, triggered by hormonal changes, stress, food, or weather shifts. With their longer duration, migraines require immediate relief and preventive strategies. Proper identification of each condition allows for better management and improved quality of life for sufferers. Despite various treatments, many patients experience insufficient relief or intolerable side effects.^60^

Psychedelics, particularly substances like psilocybin and LSD, have emerged as potential alternatives for managing CH. Preliminary evidence suggests that psychedelics may reduce the frequency and severity of attacks, possibly through their interaction with 5-HT_2A_Rs, which play a role in pain modulation and vasoconstriction.^61^ Case reports and small studies indicate that even subhallucinogenic doses can be effective.^62^

Sewell et al. retrospectively evaluated the therapeutic effects of psilocybin and LSD in 53 individuals with CH (32 episodic, 21 chronic) recruited through support groups and surveys. Participants reported acute attack termination, cluster period disruption, and remission prolongation; medical records and diaries confirmed these data. Psilocybin terminated attacks within 20 min in 85% of users and stopped cluster periods in 52% of cases, whereas LSD was effective in terminating cluster periods in 88% of cases. Both substances prolonged remissions (psilocybin: 91%; LSD: 80%), and efficacy was observed at subhallucinogenic doses in 42% of users. These results underscore the potential of psilocybin and LSD as novel treatments for CHs, warranting further investigation with controlled trials.^59^

Another study analyzed 496 verified responses from an online survey, examining the efficacy of conventional and alternative treatments for CHs, including indoleamine hallucinogens. Participants reported on abortive and preventive treatments, as well as remission effects. High-flow oxygen and subcutaneous injections of triptans were the most effective remitting methods, while psilocybin showed comparable efficacy to oxygen and was superior to oral and intranasal triptans. Psilocybin and LSD provided moderate to complete prevention of attacks in over 70% of users and were more effective than verapamil, steroids, and melatonin. Psilocybin and LSD were also associated with shortened cluster periods and transitions from chronic to episodic states with infrequent dosing. Hallucinogens were well tolerated, with mild gastrointestinal and central effects.^63^

A survey was also conducted to investigate the use of illicit drugs by Italian patients with CH. An online survey was conducted among 54 patients with CH for whom conventional treatments, including subcutaneous Sumatriptan, oxygen therapy and prophylactic drugs, were not fully effective. The respondents included people with chronic or drug-resistant CH who used various illicit substances, including psilocybin and lysergic acid derivatives (LSA, LSD). Hallucinogens, especially psilocybin, showed high perceived efficacy (77.8%) even at subhallucinogenic doses.^64^

Similar results were obtained in another study of alternative self-treatment methods for headaches and migraines, analyzing discussions on Internet forums (Shroomery.org, Bluelight.org, and Clusterbusters.org). Analysis of 411 coded items from posts showed that users showed minimal interest in psychoactive effects, often avoiding them at subpsychoactive doses. Psilocybin, LSD, and related psychedelic tryptamines were generally effective in both acute and prophylactic treatment of CHs and migraines, whereas cannabis produced more variable results. No serious adverse events were reported.^65^

A cross-sectional study was also conducted comparing drug use among 643 Dutch CH patients with the general population. Based on completed questionnaires and statistical data from Statistics Netherlands, lifetime drug use was higher in the CH group (31.7% vs 23.8%; *p* < 0.001), with increased use of cannabis, cocaine, amphetamines, psilocybin, and LSD. The results of this study showed that psilocybin and LSD reduced the frequency of CH attacks (56–60%), while attack duration was comparably shorter with psilocybin (46%).^66^

Johnson et al. studied the effects of psilocybin on headache incidence in 18 healthy volunteers in a double-blind, crossover design. They tested doses from 0 to 30 mg/70 kg and found that headache incidence, duration, and severity increased dose-dependently, with delayed onset and transient duration, resolving within 24 h. All reported headaches were mild to moderate, suggesting manageable side effects. Proposed mechanisms included nitric oxide release and modulation of serotonin pathways, mainly via the dorsal raphe nucleus. The results further contribute to understanding psilocybin side effects that are not severe enough to hinder further investigation.^67^

Another exploratory randomized, double-blind, placebo-controlled exploratory Phase 1 study (NCT02981173) evaluated the effects of low-dose psilocybin (0.143 mg/kg) versus placebo on CH suppression. Sixteen participants were randomized to receive either psilocybin or placebo in a pulse regimen of three doses spaced 5 days apart, with 14 completing the study. Headache diaries documented attack frequency, duration, and severity 2 weeks before and 8 weeks after treatment. While psilocybin reduced weekly cluster attacks, the reduction was more pronounced in chronic CH than in episodic ones. No significant differences were observed in attack duration or severity, and the reduction in attack frequency was independent of acute psychotropic effects. Psilocybin was well-tolerated, with transient adverse events, including nausea, fatigue, and anxiety. Although statistical significance was not achieved, the findings suggest psilocybin may reduce CH frequency, particularly in chronic cases, warranting further studies in larger, more diverse populations.^68^ In the blinded extension phase of this randomized controlled trial, the effects of a repeated psilocybin pulse regimen (three doses, 5 days apart) in individuals with CH were examined. Eligible participants were invited to participate in a repeat psilocybin pulse regimen administered at least six months after their initial study involvement. Ten participants completed an extension phase, keeping headache diaries 2 weeks before and 8 weeks after treatment. Results showed a significant reduction in cluster attack frequency, a 50% reduction, regardless of response to psilocybin in the first round. Treatment was well tolerated, with no unexpected or serious adverse events. These results underscore the potential of repeated psilocybin pulses for long-term relief of CH, warranting further study of long-term safety and efficacy in larger, diverse populations.^69^ The role of tryptamine derivatives in CHs is still a cause for ongoing clinical trials. For example, a clinical trial of LSD targeting patients with CHs recently began recruiting (NCT03781128).

The literature includes both clinical studies and case reports that describe a reduction in migraine frequency following the use of psychedelic substances. An example of a case study examines the acute therapeutic potential of psilocybin for migraines with aura in a 33 year-old man with a history of episodic migraines. The patient self-administered 1.2 g of Psilocybe cubensis at the onset of a migraine aura, with results comparable to three previous conventionally treated or untreated migraines. Psilocybin treatment resulted in marked reductions in headache intensity (Numerical Rating Scale 0–2 vs 6–9) and vomiting episodes (1 vs approximately 4), with no side effects reported. In contrast, prior treatments, including sumatriptan, naproxen, and acetaminophen, provided limited relief. Although promising, this single observational case with self-reported outcomes underscores the need for rigorous randomized controlled trials to confirm efficacy, safety, and precise mechanisms of action.^70^

A double-blind, placebo-controlled Phase 1 clinical trial (NCT03341689) was conducted to investigate the effects of oral psilocybin on migraine headaches.^71^ The study included ten adults with at least two migraine attacks per week, no major medical or psychiatric conditions, and no recent use of serotonergic medications. Participants attended two sessions, 2 weeks apart, receiving either placebo or psilocybin in random order. Outcomes were assessed using headache diaries alongside psychological and physiological measures. Results revealed that psilocybin significantly reduced weekly migraine days, attack frequency, pain severity, and functional impairment during migraines compared to the placebo. No serious adverse events occurred, and transient side effects such as light-headedness and muscle tension resolved without intervention. The findings suggest that a single low dose of psilocybin can significantly reduce migraine burden over 2 weeks, with therapeutic effects potentially independent of acute psychotropic experiences. This study highlights psilocybin’s promise as a novel migraine treatment, warranting further research into its mechanisms and long-term safety.

Another recent clinical trial investigated the potential of psilocybin as a therapeutic agent for relieving migraine headaches (NCT04218539). The trial included 18 participants, aged 21 to 65 years, and evaluated both single and repeated doses of psilocybin, with a maximum of two administrations. The study aimed to explore psilocybin’s mechanism of action by measuring neuroinflammatory markers associated with migraine pathophysiology. Additionally, changes in migraine attack frequency were assessed over a two month period. The findings of the study are yet to be published.

Primary headache disorders also include SUNHA (Short-lasting Unilateral Neuralgiform Headache Attacks), a rare condition characterized by brief, but frequent unilateral pain accompanied by cranial autonomic symptoms such as eye redness, tearing, nasal congestion, or ptosis. In a Phase 1b open-label study (NCT04905121), four participants received escalating doses of oral psilocybin (5 mg, 7.5 mg, 10 mg) across three sessions. Two experienced a >50% reduction in attack frequency, though improvements in duration and severity varied. Significant psychedelic effects were reported, but cognitive assessments were incomplete. No serious adverse events occurred; nausea and vivid dreams were the most common side effects. These findings suggest that psilocybin may help reduce attack frequency, warranting further study.^72^

Other clinical trials are also underway to investigate the use of psilocybin and LSD in the treatment of cluster headache disorders. A Phase 2 study (NCT05477459) is currently recruiting participants to evaluate the effects of LSD on the frequency and intensity of CH attacks. In parallel, a Phase 1/2 trial (NCT04280055), which aimed to assess the safety and efficacy of psilocybin for the same condition, has been terminated and is no longer recruiting. In the case of migraine, a new Phase 1 trial (NCT06464367) is actively recruiting and investigates the effects of single doses of psilocybin, aiming to further understand its therapeutic potential and mechanisms of action. Additionally, a Phase 1 study (NCT03806985) analyzing psilocybin’s effects on postconcussion headache has also been terminated and is no longer enrolling participants. All clinical trial data on the use of psychedelics for various types of headaches are summarized in Table 3.

**Table 3:** Clinical Trials on Classic Psychedelics for Various Types of Headache Disorders

disease	compound	Phase	clinicalTrials.gov identifier	psychedelic oral dosages and frequency
cluster headache	psilocybin	Phase 1	NCT02981173	0.143 mg/kg or 10 mg/0.0143 mg/kg or 1 mg, 3 doses, 5 days apart
	LSD	Phase 2	NCT03781128	100 μg, 3 doses within 3 weeks
	LSD	Phase 2	NCT05477459	25 μg every 3 days for 3 weeks
	psilocybin	Phase 1/2	NCT04280055	0.14 mg/kg in 3 sessions spaced by 1 week
migraine	psilocybin	Phase 1	NCT03341689	0.143 mg/kg/0.0143 mg/kg, single dose; crossover with placebo after 2 weeks
	psilocybin	Phase 1	NCT04218539	10 mg, single dose; crossover with placebo after 1 week
	psilocybin	Phase 1	NCT06464367	10 mg, frequency not specified
concussion headache	psilocybin	Phase 1	NCT03806985	0.143 mg/kg or 10 mg/0.0143 mg/kg or 1 mg, 2 doses, 14 days apart
short-lasting unilateral neuralgiform headache attacks (SUNHA)	psilocybin	Phase 1	NCT04905121	ascending doses of 5, 7.5, and 10 mg within 11 days, interval not provided

A review of registered clinical trials involving psychedelic substances in the context of headache disorders demonstrate psilocybin as the most researched compound, accounting for 7 out of 9 studies. In comparison, LSD has been included in only two studies, while DMT and mescaline have not been investigated, highlighting psilocybin’s predominant role in current clinical trials. Headache related studies exhibited great variability in both dose and administration frequency, including weight-adjusted dosing schemes (e.g., 0.143 mg/kg), ascending dose protocols (e.g., 5–10 mg over several days), and crossover designs with single, double or triple dosing schedules. LSD, the most potent 5-HT_2A_ agonist among the classical psychedelics, was administered in microgram doses, in contrast to the milligram-range doses of psilocybin (up to 10 mg) used in clinical studies. The limited scope of studies on other psychedelics emphasizes their investigational phase in pain management. These studies suggest that low-dose psychedelic regimens may offer therapeutic benefits across various headache disorders, with dosing strategies tailored to each condition.

### 4.2 Chronic Pain

Chronic pain, which persists for over three months, is a significant public health concern, affecting approximately 20% of adults worldwide.^73^ It is more prevalent among older adults, women, and individuals with lower socioeconomic status.^74^ Common types of chronic pain include lower back pain, osteoarthritis, neuropathic pain, and fibromyalgia, all of which contribute significantly to disability, reduced quality of life, and increased healthcare costs.^75^ Chronic pain can result from musculoskeletal disorders (e.g., arthritis, back pain), neuropathic injuries (e.g., diabetic neuropathy, postherpetic neuralgia), or central nervous system dysfunction (e.g., fibromyalgia, migraines). Additionally, unresolved acute pain, persistent inflammation, nerve damage, and psychological factors such as anxiety and depression can exacerbate pain perception and prolong its persistence.

Chronic pain results from persistent activation and maladaptive changes in the nervous system, involving peripheral and central sensitization.^76,77^ Peripheral sensitization occurs when prolonged injury or inflammation leads to hypersensitivity of nociceptors, increasing pain perception, which is mediated by inflammatory molecules such as prostaglandins, bradykinin, and cytokines. Central sensitization involves hyperexcitability of neurons in the spinal cord and brain, reducing pain inhibition and amplifying pain signals. Dysregulation of glutamate, substance P, and BDNF contributes to this process, leading to hyperalgesia (exaggerated pain response) and allodynia (pain from nonpainful stimuli).^78^ Additionally, chronic pain is associated with dysfunction of endogenous pain modulation systems, including the opioid and cannabinoid pathways, resulting in inadequate pain suppression. Alterations in the limbic system contribute to emotional and cognitive pain modulation, often linking chronic pain to depression and anxiety. These mechanisms sustain pain persistence.

A multimodal approach is essential for chronic pain management, integrating pharmacological and nonpharmacological therapies. Medications include NSAIDs, opioids (for severe cases), antidepressants (e.g., amitriptyline, duloxetine), and anticonvulsants (e.g., gabapentin, pregabalin).^79^ Nondrug therapies such as physical therapy, cognitive-behavioral therapy, acupuncture, and neuromodulation (e.g., spinal cord stimulation) have shown effectiveness.^80,81^

Emerging approaches, including medical psychedelic-assisted therapy, are being investigated for their potential for chronic pain relief. Psychedelic substances such as LSD and psilocybin have shown potential for chronic pain conditions such as cancer pain, fibromyalgia and phantom limb pain. Emerging clinical trials, although limited, suggest that these substances may provide significant pain relief, as well as additional benefits for depression, anxiety, and sleep disorders. Mechanistic studies indicate that these effects may be due to increased neuroplasticity, reversal of central sensitization, and anti-inflammatory effects.^71,82^ Psychedelics may impact descending pain modulation pathways, reducing the amplification of pain signals and counteract hyperactivity in brain regions involved in chronic pain persistence. Psychedelics, through 5-HT_2A_R agonism, show potential to reset functional brain connectivity, offering a promising avenue for chronic pain management, warranting further research amidst the opioid crisis.^7^ Interestingly, the analgesic effects of psychedelics might be distinct from their psychoactive experiences, suggesting a pathway for pain relief independent of perceptual alterations.^7^ While psychedelic substances have shown a favorable safety profile under controlled conditions, concerns such as cardiovascular risk and potential psychological distress require cautious handling. These substances represent a potentially revolutionary approach to pharmacological pain management, but further robust clinical trials are needed to confirm their therapeutic efficacy.^82^ Historical and more recent research confirms that psychedelic substances can relieve chronic inflammatory pain. Early work by Kast and Collins highlighted LSD’s (100 μg) superior pain relief compared to opioids in 50 severely ill patients, hypothesizing that the analgesic LSD’s effects stem from altered pain attention and perception.^83^ Over the past decade, classic psychedelics have been extensively researched for their potential to treat chronic pain, as evidenced by population-based studies, case reports, and clinical trials described below.

Bornemann et al. conducted a qualitative study on self-medication with classic psychedelics for chronic pain, involving 11 participants in semistructured interviews. The study found significant pain reductions during and after psychedelic use, alongside positive reframing of pain and enhanced somatic awareness. Complementary practices like mindfulness and movement supported these outcomes. Despite limitations, such as a small, biased sample and retrospective data, the study offers valuable insights for clinical trial design, emphasizing patient-centered approaches and mechanisms like cognitive reframing and embodiment. These findings suggest psychedelics’ transformative potential in pain management and underscore the need for controlled trials and mechanistic studies.^84^

Similarly, Bonnelle et al.^85^ conducted a double-blind study on the efficacy of psychedelics (psilocybin, LSD, ayahuasca, DMT, and mescaline) for managing chronic pain, comparing macrodoses (hallucinogenic) and microdoses (subhallucinogenic) to conventional pain medications. An online survey of 250 chronic pain sufferers (ages 31–40) assessed pain relief, side effects, and satisfaction. Statistical analyses showed macrodoses provided the most significant pain relief, outperforming microdoses, opioids, and over-the-counter drugs. Macrodoses also offered longer-lasting relief, with 33.7% experiencing benefits for over 3 days, compared to 21.4% for microdoses. Microdoses caused fewer side effects than conventional medications but were less effective than opioids. Notably, macrodoses significantly improved life satisfaction and reduced pain interference, while microdoses were more effective when pain relief was the primary goal. These results highlight psychedelics’ potential, especially macrodoses, as promising analgesic alternatives, warranting further research into clinical validation, optimal dosing, and mechanisms of action.

Another cross-sectional online study^86^ of 354 North Americans with fibromyalgia explored their knowledge, perceptions, and use of serotonergic (e.g., psilocybin, LSD, DMT) and nonserotonergic psychedelics (e.g., ketamine), as well as interest in psychedelic-based therapies. Nearly 30% had used psychedelics, primarily LSD and psilocybin. Most perceptions were neutral (59.4%) or positive (36.8%), with under 3% reporting negative effects on health or pain. Among 12 participants using psychedelics for pain relief, 11 reported symptom improvement. Regardless of prior use, most participants supported psychedelics’ potential for treating fibromyalgia and expressed interest in clinical trials. These findings emphasize the need for further research into psychedelics as a treatment for fibromyalgia symptoms.

Ramachandran et al.^87^ reported a patient using psilocybin (microdoses 0.2–0.5 g and macrodoses: 2–3 g) during multiple sessions for phantom limb pain, experienced significant pain reduction for 3 weeks, along with a decrease in paroxysmal episodes for the same duration. These effects were greater than those achieved with mirror-visual feedback (MVF) and “phantom massage” alone. In another case, the therapeutic potential of microdosed psilocybin for chronic pain in three patients with neuropathic and musculoskeletal disorders was investigated.^88^ Patients experienced significant pain relief (80%–100%) with doses ranging from 250 mg to 1 g of dried mushrooms, accompanied by improved functional capacity and minimal side effects. Synergistic benefits were noted when combined with physical therapy, and the effects lasted up to 4 weeks without evidence of tolerance or dependence. The analgesic effects were attributed to activation of the 5-HT_2A_R, suggesting central modulation of nociception and synaptic plasticity.

Ramaekers et al.^89^ investigated the analgesic effects of low-dose LSD in a randomized, double-blind, placebo-controlled crossover trial involving 24 healthy volunteers. Participants received single doses of 5, 10, and 20 μg LSD or placebo, with pain tolerance assessed via the Cold Pressor Test (CPT) and additional evaluations of subjective pain ratings, dissociative states, psychiatric symptoms, and vital signs. The 20 μg dose significantly increased pain tolerance and reduced pain intensity and unpleasantness, with a trend toward reduced unpleasantness at 10 μg. Mild psychological effects and modest increases in blood pressure were observed at 20 μg but remained clinically insignificant. Plasma LSD levels correlated with dose and sustained effects for up to 5 h. These findings suggest that low-dose LSD offers a promising analgesic alternative with minimal psychedelic effects, warranting further investigation in clinical populations.

Psilocybin-assisted therapy represents a novel approach for treating fibromyalgia, a chronic pain syndrome characterized by widespread pain, sleep disturbances, and mood challenges. In this Phase 2 open-label pilot trial (NCT05128162),^90^ five adults with fibromyalgia received two oral psilocybin doses (15 mg and 25 mg) in conjunction with psychotherapy. Safety outcomes revealed no serious adverse events, with only transient side effects such as mild headaches. Clinically significant improvements were observed in pain severity, interference, sleep disturbances, and enhanced psychological resilience and emotional well-being. While the results are promising, the small sample size and absence of a control group limit generalizability. This study highlights the potential of psilocybin-assisted therapy for fibromyalgia and underscores the need for larger, randomized trials to confirm efficacy, safety, and mechanistic pathways.

Several ongoing clinical trials are exploring the potential of psilocybin in managing chronic pain and related conditions. Among these, Phase 2 (NCT04950608) is a pilot study examining psilocybin-assisted therapy for demoralisation in hospice care, focusing on alleviating psychological and existential distress in terminally ill patients. An observational study (NCT05548075) investigates electroencephalogram (EEG)-based brain biomarkers of psilocybin’s effects on individuals with fibromyalgia. Additionally, NCT05305105 (Phase 1) evaluates psilocybin’s impact on symptom burden and quality of life in post-treatment Lyme disease, while NCT05506982 (Phase 1) examines its use in combination with palliative care for cancer survivors experiencing chronic pain and demoralisation. Recruiting studies also investigate psilocybin’s therapeutic potential for various chronic pain conditions. NCT05068791 (Phase 1) focuses on its efficacy in fibromyalgia, aiming to reduce symptoms such as pain and fatigue while improving quality of life and identifying treatment mediators. NCT05224336 (Phase 1) is a double-blind, placebo-controlled study assessing psilocybin’s safety and effects on pain, mood, and neural responses in individuals with phantom limb pain. Furthermore, NCT06368492 examines psilocybin’s impact on pain and quality of life in fibromyalgia, NCT05351541 (Phase 1/2) evaluates its therapeutic potential for chronic low back pain, and NCT06355414 (Phase 1) investigates its dual effects on pain and depression in patients with chronic back pain. Additional Phase 1 and Phase 2 trials have recently been registered, including NCT06919640 (Phase 1), investigating psilocybin for general chronic pain, and NCT06001749 (Phase 2), evaluating its efficacy in managing cancer-related pain. Another ongoing study, NCT06827054 (Phase 2), targets chronic pain in cancer patients specifically, expanding the evidence base for psilocybin’s role in oncology-related symptom management. Upcoming Phase 2 trials include NCT06731335, which aims to evaluate psilocybin’s role in enhancing analgesia for chronic neuropathic pain, and NCT06518720, targeting treatment-resistant depression and chronic neuropathic pain as part of the TRANSCEND study. Additionally, NCT05585229 will explore psilocybin-assisted psychotherapy as a strategy for tapering opioid medication in the management of chronic pain. Clinical trials are being conducted not only with psilocybin but also with LSD. Recruitment is also ongoing for the Phase 2 study (NCT05883540) on the use of LSD in Palliative Care (LPC). Additionally, a randomized, placebo-controlled trial (NCT06180759) is underway, investigating the acute analgesic effects of DMT on experimentally induced pain in healthy participants comparing its efficacy to racemic ketamine and placebo. Table 4 summarizes clinical trial data on the use of psychedelics for various chronic pain disorders.

**Table 4:** Clinical Trials on Classic Psychedelics for Various Chronic Pain Disorders

disease	compound	Phase	clinicalTrials.gov identifier	psychedelic dosages and frequency
phantom limb pain	psilocybin	Phase 1	NCT05224336	25 mg (oral), single dose
palliative care	LSD	Phase 2	NCT05883540	100 μg and 100 μg or 100 μg and 200 μg/25 μg and 25 μg (oral), dosing frequency not specified
lyme disease	psilocybin	Phase 1	NCT05305105	15 mg and 25 mg (oral), two doses, 2 weeks apart
chronic pain^a^	psilocybin	Phase 2	NCT05585229	25 mg and 37.5 mg (oral), two doses, month apart
chronic low back pain	psilocybin	Phase 1	NCT06355414	25 mg (oral), single dose
chronic neuropathic pain	psilocybin	Phase 2	NCT06731335	25 mg (oral), single dose
chronic neuropathic pain and depression	psilocybin	Phase 2	NCT06518720	25 mg (oral), single dose
chronic low back pain	psilocybin	Phase 1/2	NCT05351541	single oral dose: 1 mg-30 mg in combination with drug(s) (zolpidem or modafinil or zolpidem and modafinil) or with placebo
chronic pain in cancer survivors	psilocybin	Phase 1	NCT05506982	25 mg (oral), single dose
cancer pain treatment	psilocybin	Phase 2	NCT06001749	dose/frequency not specified
chronic pain	psilocybin	Phase 1	NCT06919640	10 mg, single dose
chronic pain in cancer patients	psilocybin	Phase 2	NCT06827054	8 doses in 4 weeks, dose not specified
chronic pain, migraine, cluster headache or phantom limb pain	DMT	Phase 1	NCT06180759	1.2 mg/min (infusion), single dose
fibromyalgia	psilocybin	Phase 2	NCT05128162	15 mg and 25 mg (oral), two doses, 2 weeks apart
	psilocybin	Phase 1	NCT05068791	0.36 mg/kg (oral), single dose
	psilocybin	not applicable	NCT06368492	5 mg and 10 mg (oral), two doses; crossover with placebo, dosing frequency not specified
	psilocybin	observational	NCT05548075	up to 25 mg (oral), two doses, a month apart
terminal illness in hospital care	psilocybin	Phase 2	NCT04950608	25 mg (oral), single dose

a Tapering long-term opioid therapy in chronically ill patients using psilocybin-assisted psychotherapy.

An analysis of registered clinical trials exploring the use of classic psychedelic substances for pain management reveals that psilocybin is the most extensively studied compound, featured in 11 out of 18 chronic pain trials. In contrast, LSD and DMT appear in only one chronic pain study each, underscoring psilocybin’s dominant role in current research. Dosing approaches varied considerably, though a single oral dose of 25 mg psilocybin emerged as the most frequently used regimen, employed in six separate trials, suggesting a movement toward standardized fixed-dose protocols. Conversely, LSD was typically administered in microdoses (e.g., 25 μg) or low doses (e.g., 100 μg), and DMT was used in low-dose intravenous infusions (e.g., 1.2 mg/min). The relatively small number of studies involving psychedelics other than psilocybin, only two of the 18 total, highlights their early stage status in pain research and further reinforces psilocybin’s central position in this emerging field.

## 5 Futures Directions and Conclusion

The therapeutic landscape of chronic pain is evolving, with increasing interest in psychedelic-assisted therapy as a potential alternative to conventional pain management strategies. While current treatment options such as opioids, NSAIDs, and neuromodulation techniques offer relief, they often fall short due to side effects, tolerance, dependency risks, and limited long-term efficacy. The opioid crisis underscores the urgent need for nonaddictive and effective alternatives, making psychedelics a promising research avenue.

The therapeutic potential of classic psychedelics like psilocybin and LSD is limited by their hallucinogenic effects, driving interest in nonhallucinogenic psychedelic analogues. One such compound is 2-bromo-LSD (2-Br-LSD, BOL-148), a nonhallucinogenic LSD derivative that acts as a partial agonist at multiple aminergic GPCRs, including 5-HT_2A_ but lacks 5-HT_2B_ agonism, reducing cardiac risk and preventing tolerance buildup with repeated use. Preclinical studies reveal high affinity binding to 5-HT_2A_ (*K*_i_ = 0.5–2.2 nM), where it acts as a partial agonist through both Gq and β-arrestin 2 (βarr2) pathways, with EC_50_ values of 0.81 nM and 0.73 nM, respectively. At 5-HT_1A_, 2-Br-LSD shows partial agonist activity via G protein signaling (EC_50_ = 11.3 nM), but antagonism in the βarr2 pathway (*K*_b_ = 155 nM), indicating functional selectivity. Notably, 2-Br-LSD’s antagonism of 5-HT_2B_ receptors in both Gq and βarr2 pathways (*K*_b_ = 3.71 nM and 3.09 nM) prevents the valvulopathy risk linked to 5-HT_2B_ agonism seen in LSD. In addition, the compound exhibits partial agonism at 5-HT_2C_ (*K*_i_ = 19 nM, EC_50_ = 3.85 nM via Gq), with antagonistic activity in the βarr2 pathway (*K*_b_ = 2.64 nM), suggesting a nuanced profile relevant to mood and cognition regulation. Beyond the serotonin system, 2-Br-LSD also engages dopaminergic receptors, functioning as a D_1_ antagonist (*K*_i_ = 25 nM) and a D_2_ agonist (*K*_i_ = 2.1–4 nM), and interacts with α_1A_ adrenergic receptors as an antagonist (*K*_i_ = 59–250 nM). Notably, it does not strongly engage histaminergic receptors (H_1_), further distinguishing its profile from LSD.^91^ In a preliminary open-label, nonrandomized case series^81^ five male patients (aged 28–47 years) with severe, treatment-resistant CH received three oral doses of 2-Br-LSD (30 mg/kg) over 10 days. Four patients experienced substantial improvements: one had a complete remission for six months, two transitioned from chronic to episodic CH with remissions lasting 1–9 months, and one reported reduced attack frequency. The fifth patient experienced a 30% reduction in pain intensity but no change in attack frequency. Mild, transient side effects occurred without hallucinogenic effects or changes in vital signs. These findings suggest that 2-Br-LSD may effectively interrupt CH cycles and induce prolonged remission with minimal adverse effects. However, more extensive randomized controlled trials are needed to confirm its efficacy and safety.^81^ A promising alternative, 2-Br-LSD, a nontoxic, and nonhallucinogenic LSD derivative, has shown potential to prevent CH attacks, offering a safer option for research and clinical use.

In addition to 2-Br-LSD, nonhallucinogenic compounds derived from the ibogaine scaffold, known as ibogalogs, represent a novel and increasingly relevant area of research in pain therapy. These compounds exhibit serotonergic or kappa–opioidergic activity and have demonstrated analgesic potential in preclinical models of neuropathic and inflammatory pain. Several ibogaine derivatives, such as tabernanthalog (TBG), ibogaminalog (DM506), oxa-noribogaine, and benzofuran analogs, have shown analgesic effects through diverse receptor mechanisms, including 5-HT_2A_, mGlu2, and kappa opioid receptors (KOR). Using established mouse models of chronic pain, studies have shown that TBG, ibogainalog (IBG), and DM506 effectively alleviate mechanical and thermal hypersensitivity. These antinociceptive effects were fully reversed by the 5-HT_2A_ receptor antagonist ketanserin, confirming a serotonergic mechanism of action.^91^ Among these, IBG produced the longest-lasting effects, while DM506 showed the fastest onset of action. In an oxaliplatin-induced neuropathic pain model, catharanthalog (CAG), noribogainalog (nor-IBG), and another ibogalog derivative, PNU-22394 also demonstrated consistent analgesic efficacy without observable toxicity.^92^ Interestingly, both IBG and nor-IBG required coadministration with the mGlu2 agonist LY379268 at subtherapeutic doses, suggesting a synergistic mechanism involving 5-HT_2A_–mGlu2 receptor crosstalk. Concurrently, oxa-iboga analogs have emerged as nonhallucinogenic compounds with potent analgesic activity mediated primarily through KOR pathways. Both oxa-noribogaine and its stereoisomer, epi-oxa-noribogaine, produced pronounced antinociceptive effects in the tail-flick assay, which were abolished in KOR-deficient mice, confirming a KOR-dependent mechanism.^93^ Similar analgesic and anti-inflammatory effects were observed for benzofuran ibogalogs in the formalin pain model. These compounds not only reduced nociceptive behaviors but also reversed neuroinflammatory markers such as substance P, CGRP, and COX-2, while restoring levels of neurotrophic factors like BDNF and GDNF.^94^ Despite the promising preclinical data, clinical evidence is currently lacking. No completed or ongoing clinical trials targeting ibogalogs, such as TBG, IBG, DM506, or oxa-ibogaine, are registered in public databases. The investigation of ibogaine analogs as nonhallucinogenic pain therapeutics represents an innovative and underexplored frontier in psychedelic science. While serotonergic psychedelics like psilocybin and LSD are increasingly studied for their potential in pain modulation, ibogalogs provide a distinct, nonhallucinogenic approach with demonstrated preclinical efficacy.

Emerging clinical trials investigating psychedelics, particularly psilocybin and LSD, suggest they may provide long-lasting, analgesic effects, independent of their hallucinogenic properties. Preclinical and early phase studies indicate that 5-HT_2A_R agonism, neuroplasticity enhancement, and anti-inflammatory properties may underlie their potential analgesic effects.

Another major limitation in psychedelic clinical trials is the difficulty of maintaining effective double-blind conditions. Substances such as psilocybin, DMT, mescaline, and LSD have profound and distinctive psychoactive effects, often making it easy for participants and sometimes researchers to identify whether they have received the active drug or a placebo.^95^ Although strategies such as using active placebos (e.g., niacin or subthreshold psychedelic doses) and alternative trial designs have been proposed, available evidence suggests that these approaches are only partially effective. Moreover, blinding assessments remain infrequently reported in clinical studies,^96^ and the relational and contextual nature of psychedelic therapy, characterized by heightened suggestibility and the therapeutic setting, further complicates attempts to isolate pharmacological effects from psychological or interpersonal factors.^97^ Small sample sizes, selection bias, short-term follow-up, and other validity concerns threaten the construct and statistical conclusion validity of psychedelic research.^98^ The development and application of nonhallucinogenic psychedelics may offer a promising approach to overcoming some of these methodological barriers by reducing the risk of functional unblinding while preserving potential therapeutic effects. As a result, there is growing consensus that clinical research on psychedelics requires improved methodological rigor, including standardized assessment of blinding integrity, formal measurement of participant expectancy, and greater transparency in trial design and reporting practices. Acknowledging and addressing these challenges is essential for producing valid, reproducible, and clinically meaningful findings in psychedelic research.

Subhallucinogenic (microdose) regimens have become a key focus of interest, as they may offer therapeutic benefits without the perceptual distortions typically associated with psychedelics, potentially improving clinical acceptability. So far, controlled studies have suggested that microdosing psychedelics like LSD is generally safe and may produce mild effects on mood, pain perception, and brain activity.^99^ However, evidence of long-term benefits remains limited. PET imaging in healthy volunteers showed that psilocybin doses between 3 and 30 mg occupied 43–72% of 5-HT_2A_ receptors 1 h after ingestion, with occupancy decreasing over time. A subperceptual dose (10 mg/70 kg) produced an average occupancy of approximately 39.5%.^100,101^ To date, no PET studies have assessed 5-HT_2A_ receptor occupancy for LSD or mescaline in humans. While microdosing appears promising, its clinical potential remains unproven and requires further investigation, particularly in patient populations. Additionally, psychedelic-assisted psychotherapy is being explored for its ability to address the emotional and cognitive aspects of chronic pain, including pain-related catastrophising, anxiety, and depression, which often exacerbate pain perception. Thus, future research should focus on large-scale randomized controlled trials (RCTs) to confirm efficacy and safety. Next, long-term studies will assess the sustainability of analgesic effects, and mechanistic studies will explore interactions with pain modulation networks, neuroplasticity, and inflammatory pathways. As research progresses, collaborations between neurologists, pain specialists, psychiatrists, and regulatory agencies will be essential to translate psychedelics into viable clinical treatments for chronic pain conditions. Moreover, regulatory challenges and societal perceptions also necessitate educational initiatives and policy reforms to pave the way for responsible medical use.

In conclusion, psychedelic-assisted therapy represents a paradigm shift in pain treatment, offering a multidimensional approach that targets both the sensory and emotional aspects of pain. As scientific and clinical research advances, psychedelics may reshape the future of pain medicine, providing effective, nonaddictive, and long-lasting relief for millions suffering from different types of chronic pain worldwide.

## Abbreviations

5-HT: serotonin
5-HT_2A_R: serotonin 2A receptors
AMPA: α-amino-3-hydroxy-5-methyl-4-isoxazolepropionic acid receptor
BBB: blood–brain barier
BDNF: brain-derived neurotrophic factor
CaMKII: calmodulin-dependent protein kinase
cAMP: cyclic adenosine monophosphate
CH: cluster headache
CL: clearance
*C*_max_: maximum concentration
CNS: central nervous system
CPT: cold pressor test
CREB: element-binding
DAG: diacylglycerol
d.e: duration effect
DH: dorsal horn
DMT: *N*,*N*-dimethyltryptamine
DOI: 2,5-dimethoxy-4-iodoamphetamine
DR: dopamine receptor
EEG: electroencephalogram
EFNS: European Federation of Neurological Societies
ERKs: extracellular signal-regulated kinases
GPCR: G-protein-coupled receptor
HR: histamine receptor
IASP: International Association for the Study of Pain
IL-6: interleukine-6
IP_3_: inositol trisphosphate
LPC: LSD in palliative care
LSD: lysergic acid diethylamide
LTP: long-term potentiation
mTOR: mammalian target of rapamycin
MAO: monoamine oxidase
MVF: mirror-visual-feedback
NMDA: *N*-methyl- *d* -aspartate
NSAIDs: nonsteroidal anti-inflammatory drugs
PAG: periaqueductal gray
PKC: protein kinase C
PLC&gamma: phospholipase C gamma
PNS: peripheral nervous system
PTSD: posttraumatic stress disorder
RCT: randomized controlled trials
RVM: rostroventral medulla
TAC: trigeminal autonomic cephalalgias
*T*_max_: time to maximum
TNF-&alpha: tumor necrosis factor α
TrkB: tropomyosin receptor kinase B
*T*_1/2_: terminal elimination half-time
αR: adrenergic receptor
